# Validation and design of a wearable harness for monitoring remote heart rate variability in dairy cows under grazing conditions

**DOI:** 10.1007/s11250-026-04859-4

**Published:** 2026-02-02

**Authors:** Patricia Betancourth–Chaves, John Montoya–Zuluaga, Berardo Rodríguez, Andrés Timarán–Rivera, Lina Ríos–Peña, Filadelfo Hernández–Oviedo, Darío Vallejo–Timarán

**Affiliations:** 1 Veterinary Medicine and Animal Sciences Faculty, GISCA Research Group, Institución Universitaria Visión de las Americas, Medellín, Colombia; 2 Agrarian Science Faculty, School of Veterinary Medicine, University of Antioquia, Medellín, Colombia; 3 Obonuco Research Center, Corporación Colombiana de Investigación Agropecuaria – AGROSAVIA, Pasto, Colombia

**Keywords:** Dairy cattle, High-tropics, Heart rate variability, Animal welfare

## Abstract

In veterinary medicine, heart rate variability (HRV) has emerged as a valuable tool for assessing welfare, comfort, stress responses, and overall physiological status. This study aimed to design and validate a harness/vest wearable system for remote HRV measurement and monitoring in dairy cows managed under rotational grazing conditions in the high tropics. The harness/vest was developed to protect and stabilize Holter-type measurement devices under field conditions, ensuring that it did not interfere with animal comfort and allowed proper HRV signal acquisition. For HRV measurements, a Polar H10^®^ Heart Rate Sensor paired with a Polar Unite^®^ watch was used. To evaluate the practical application of the harness/vest and its impact on animal comfort, lactating Holstein and Holstein x Jersey crossbreed dairy cows (*n* = 74) were monitored. The behavior of the animals was observed, and these observations were subsequently compiled into an ethogram, used as an outcome measure to verify whether the harness/vest altered natural behaviors. Behavioral and physiological responses were compared between cows wearing the harness/vest and those without it. The observed behavioral patterns, including posture, locomotion, feeding behavior, and social interactions, were similar between groups, indicating that the harness/vest did not interfere with comfort or routine activities. Comfort was assessed through: (1) comparison of frequencies and durations of key behaviors, (2) evaluation of any avoidance or agitation responses at fitting, and (3) continuous observation for signs of mechanical interference, rubbing, or restraint. Based on these evaluations, Holter-type devices can be reliably integrated into the harness/vest system developed in this study, representing an innovative and effective tool for continuous, remote, and noninvasive HRV monitoring in grazing dairy cows in the high tropics, while preserving comfort and welfare.

**Supplementary Information:**

The online version contains supplementary material available at https://doi.org/10.1007/s11250-026-04859-4.

## Introduction

In veterinary medicine, heart rate variability (HRV) has emerged as a valuable non-invasive tool for assessing welfare, comfort, stress responses and physiological status (von Borell et al. 2007; Kovács, 2014). In dairy cattle, HRV has been applied to evaluate management practices and animal-based variables that impact welfare for example, differences in temperament (Kovács et al. 2015a, b), production and body condition (Frei et al. 2022), and pasture­based settings (Wierig et al. 2018). From a physiological perspective, HRV provides insight into autonomic nervous system regulation of cardiac activity (time-domain, frequency-domain, non-linear indices) and thus reveals internal physiological changes in response to stress or altered welfare states (von Borell et al. 2007; Kovács, 2014). Although various techniques have been developed to record and analyse cardiac activity in cattle (e.g., ECG, portable heart-rate monitors, wearables), the precision, accessibility and field feasibility of these methods vary considerably and authors highlight the need for standardized protocols, robust signal processing and artefact correction to ensure reliable cardiac physiological assessment (Wierig et al. 2018; Kovács, 2014).

Assessing cardiac physiology in dairy cows under grazing conditions in the high tropics presents environmental, management, behavioral, and technological challenges (Bun et al. 2018). Specialized dairy production in Colombia is conducted mainly on herds located on highlands with altitudes ranging from 2400 to 3200 m. In this area, dairy production is performed in high-altitude lands (2547 ± 156 m) with average temperatures of 16.3 ± 1.46 °C, herds with Holstein as the predominant breed, and manual and mechanical milking directly in barns or paddocks (Vallejo-Timarán et al. 2020). High tropical regions are characterized by variations in altitude, temperature, humidity, and particular soils and geography, which influence cow adaptation and behavior (Raza et al. 2024). Measuring HRV in grazing dairy cows presents several challenges due to environmental conditions, continuous movement, variable body postures, and limited control over sensor placement and stability. These factors can introduce motion artefacts, signal noise, electrode displacement, and loss of R-R interval accuracy, all of which compromise HRV reliability (Wierig et al. 2018; Kovács, 2014). Traditional Holter monitor configurations often require adhesive electrodes or rigid housings that are unsuitable for humid, muddy, or highly mobile grazing environments, and repeated friction with vegetation or conspecifics can further destabilize the sensors (Kovács et al. 2015a, b). The harness/vest developed in this study was specifically designed to address these limitations by providing stable fixation of the Holter sensor, preventing lateral displacement, reducing movement artefacts through textile tension control, and protecting the device from environmental exposure. This improves HRV signal quality under pasture-based conditions by maintaining consistent skin contact and minimizing interference during grazing, walking, rumination, and social interactions.

The adequate utilization of Holter measurement devices in field conditions faces a challenge; equipment such as electrocardiographs (ECGs) and heart rate monitors (HRMs) requires a proper fit to the animal’s body to obtain accurate recordings (Von Borell et al. 2007; Kahankova et al. 2022; Kim et al. 2018). However, under grazing conditions, these devices can become loose or displaced due to the constant movement of the animal, leading to artifacts and signal loss. In addition, changes in topography and uneven terrain can affect device placement and data capture. Another relevant aspect in high tropics is fluctuations in temperature, rain, and humidity (Vencloviene et al. 2023), which can interfere with the electrodes and sensors of the devices, affecting the conduction of signals and reducing the reliability of the records. Additionally, heat and prolonged exposure to sunlight can alter the adherence of sensors (Stark et al. 2016). These characteristics affect the tolerance of animals to more invasive devices, such as traditional ECGs, with remote heart rate variability monitoring being the best alternative; however, using Holter sensors under grazing conditions requires additional elements that guarantee stable placement, fixed positioning, and constant recording of the information while not interfering with the comfort and normal routines of the animals. On the basis of the above, this study aimed to design and validate a vest-type wearable textile piece for remote HRV measurement and monitoring in dairy cows under rotational grazing conditions in the high tropics. This study offers additional considerations related to the use of vest-associated Holter devices.

## Materials and methods

### Study population and experimental design

This study was carried out in the specialized dairy herd of the Obonuco Research Center of the Colombian Agricultural Research Corporation – AGROSAVIA in Pasto, Colombia. To evaluate the practical application of the harness/vest and its effects on animal comfort, a total of 74 lactating dairy cows were enrolled. The herd consisted of Holstein and F1 Holstein **×** Jersey crosses. Eligible cows were postpartum, non-pregnant, between 45 and 70 days in milk, and clinically healthy. Health status was confirmed through normal physical examinations, echocardiography, and BGEM profiles (blood gases, electrolytes, and metabolites). Data collection was performed using a Polar H10^®^ heart rate sensor (Polar Electro & Oy 2025a), with the Polar Unite^®^ watch (Polar EO 2025) serving as the receiver, meaning that no clinical Holter system was used or compared. For logistical reasons, including grazing management, handling capacity, availability of operators and equipment, and the number of available sensors, the 74 cows were divided into 10 groups, each group being evaluated under the same environmental and grazing conditions until the whole sample (*n* = 74) had been evaluated. These subgroups were not experimental treatment groups; all cows underwent exactly the same HRV monitoring protocol. Approximately 10 cows were measured each week, but not simultaneously; rather, cows within each group were fitted and recorded sequentially following the same standardized protocol. This grouping strategy ensured uniformity of measurement conditions while accommodating herd management practices and sensor availability.

### Animal selection criteria and environmental and grazing conditions

Only clinically healthy cows were included in the study. Health status was confirmed by: (i) normal physical examination, (ii) normal echocardiographic findings, and (iii) normal values for blood gases, electrolytes, and metabolites (BGEM). Cows with mastitis, lameness, fever, cardiac arrhythmias, or any systemic disease were excluded. No cows were removed during the study due to health issues or device intolerance. All HRV recordings were performed during the morning-to-afternoon grazing period under typical high-tropical conditions of the Obonuco Research Center. Average temperature ranged between 12 and 17 °C with relative humidity between 70 and 85%. Cows grazed on kikuyu (penissetum clandestinum) pastures, and followed their routine path from the milking room to the paddocks (average 300–450 m). No additional handling was performed during monitoring, and cows were allowed to move without restrictions between grazing areas.

### Vest-type textile design and HRV monitoring process

The harness (constructed with waterproof and abrasion-resistant fabric) consisted of four adjustable straps positioned anatomically around the cow’s body: (a) a thoracic strap encircling the chest from left to right; (b) a cervical strap placed around the neck; (c) a caudal strap positioned around the hips; and (d) lateral flank straps that secured the system to a central dorsal vest located over the withers, where the Polar Unite^®^ receiver was housed and protected. On the internal surface of the thoracic strap, on the left lateral thoracic region, we attached the Polar H10^®^ (Polar Electro Oy, Kempele, Finland) sensor strap using Velcro. The strap included two integrated electrodes, positioned at elbow height following the manufacturer’s anatomical specifications.

Before fitting the harness, the skin in the sensor contact area was washed with a surgical soap solution, and both electrodes were moistened and covered with a conductive adhesive EEG paste (AC-CREAM^®^) to ensure high-quality electrical contact. After the sensor was attached, we confirmed its correct functioning by verifying a stable ECG waveform and heart rate detection in real time using the Polar Flow^®^ application (Polar Electro & Oy 2025b). For the HRV evaluation, the harness containing both the sensor and the receiver was fitted to the cows immediately after morning milking, and the animals were released to pasture under routine grazing conditions. The harness was removed after afternoon milking. Although the placement and removal times were consistent, the total duration of HRV recording varied between 1 and 7 h depending on each group’s grazing route, weather constraints, and farm logistics.

HRV recordings were analyzed using Kubios HRV Scientific Premium^®^ software (Tarvainen et al. 2014). From each recording, we extracted the following time-domain, frequency-domain, and nonlinear HRV indices: mean R–R interval (MRR), SDNN, RMSSD, LF/HF ratio, and SD2/SD1. To ensure data quality, we removed artefactual segments following Kubios’ automatic correction algorithms and manually verified ECG stability. Additionally, the first 5 min (transition from chute to pasture) and the last 5 min (movement from pasture to the milking parlor and harness removal) were cropped from all recordings to avoid artefacts associated with handling. Clean segments were selected based on > 95% valid R–R intervals.

### HRV monitoring workflow

To ensure full replicability of the monitoring procedure, the HRV recording protocol followed a standardized workflow. The HRV monitoring protocol followed eight steps. (i) *Habituation*: the vest (without sensor) was placed the afternoon before evaluation to allow at least 8 h of habituation. (ii) *Sensor and recording disposal colocation*: the following morning, after the cows finished the morning milking, the sensor and watch were put into the vest. (iii) *Electrode preparation*: the thoracic band (with sensor) was wetted with water, and conductive paste was put before fastening. (iv) *Verification*: correct functioning of the ECG trace was verified via the Polar Flow app for at least 30 s. (v) *Grazing*: cows were released, walking from the milking room to the grazing pastures. (vi) *Recording*: HRV was recorded continuously until the afternoon milking. (vii) *Equipment extraction and cleaning*: the vest and equipment were removed immediately after milking. (viii) *Data extraction*: HRV parameters were downloaded to Kubios HRV Scientific (v.3.5) for cleaning and analysis.

### Vest-type textile piece technical validation and HRV recording quality criteria

The technical validation of the harness was based on internationally accepted standards for measuring heart rate variability (HRV) in large animals. It also followed the guidelines of the Task Force of the European Society of Cardiology and NASPE, which say that RR intervals must be at least 5 min long and free of artifacts for temporal and spectral HRV assessment (Task Force, 1996). Following the methodology applied in cattle by Kovács et al. (2014), 5-minute segments from each continuous recording were processed in Kubios HRV Scientific using a 0.3 threshold artifact filter and a “smoothness priors” detrending algorithm (λ = 1000), ensuring compatibility with established correction procedures for autonomic analyses. The Polar H10^®^ sensor in the harness samples at 1,000 Hz, which is the recommended sampling frequency for accurate R-wave detection. Studies in both humans and cattle show that short-term HRV indices such as RMSSD are sensitive to RR temporal resolution (Berntson et al. 2005) and reliable RR measurement in cattle depends on high-quality cardiovascular signal acquisition (Salzer et al. 2022). The physiological validity of the recorded RR intervals was substantiated by comparing their distributions to values documented in controlled bovine HRV studies, which indicate that resting dairy cows consistently exhibit RR intervals ranging from approximately 700 to 1,200 ms, contingent upon their arousal state (Kovács et al. 2014). Intervals that were longer than 1,200–1,300 ms were either fixed or left out based on standard bovine HRV filtering rules. After correcting for artifacts, the temporal (RRmean, SDNN, RMSSD) and spectral (LF, HF, LF/HF) parameters fell within the expected ranges for healthy dairy cows. They also showed autonomic patterns that were consistent with grazing and rumination behaviors. This is in line with recent reports that show HRV is a strong non-invasive indicator of bovine emotional state and welfare (von Borell et al. 2007). The harness made sure that the electrode–skin conduction stayed stable while the animal was moving. This solved one of the main problems with free-grazing HRV monitoring, which is that movement, hide thickness, and exposure to the environment often make the signal quality worse (Hopster & Blokhuis,^1994^).). These findings show that the harness meets the technical requirements for valid HRV acquisition in the field and provides a scientifically sound basis for future long-term and environmental validation studies, as the reviewer suggested.

### Vest-type textile piece behavioral validation

The behavioral evaluation was carried out in a commercial herd of 74 lactating dairy cows managed under rotational grazing in the high tropics. Because the HRV harness was deployed in 10 distinct measurement rounds, during which 7–8 cows wore the device at a time, the behavioral assessment adhered to the same structure: one behavioral evaluation per deployment session, always observing the entire herd. This approach ensured that both instrumented and non-instrumented animals shared identical environmental, social, and grazing conditions. By evaluating the full group in each session rather than isolating vested cows, the design enabled direct behavioral comparison within a socially stable herd, avoiding disruptions in hierarchy, spacing or feeding patterns.

### Behavioral observation protocol

were performed by a single trained ethologist, a lot-level live scan sampling strategy was adopted an approach widely recommended in field studies of grazing cattle where individual continuous tracking is not feasible. Each behavioral session consisted of 60 min of structured observation carried out between 11:30 and 12:30 h, a period of naturally stable grazing activity that minimizes confounding effects of milking, handling or shade-seeking. The observer conducted 20 systematic scans per session, following a fixed cycle: (a) Two minutes of uninterrupted visual scanning of the entire herd; (b) One minute of rest before the next scan. This procedure resulted in a total of 200 scans across 10 sessions, providing a robust sampling density capable of detecting subtle changes in group-level behavioral dynamics.

### Observer positioning and visual coverage

All sessions were conducted from a predefined vantage point on the paddock boundary that maximized the field of view. Only animals clearly visible during each scan were included in behavioral tallies; no attempt was made to census all individuals simultaneously. This adheres to established field-ethology guidelines, which emphasize proportional representation over exhaustive headcounts when studying large ruminant groups in open pasture.

### Ethogram and behavioral definitions

A structured ethogram was developed to encompass the core repertoire of bovine behaviors associated with comfort, welfare and natural grazing activity: 1) Positive / Normal Behaviors, (a) *Grazing*: sustained forage intake with head lowered and rhythmic bite–chew cycles; (b) *Rumination*: rhythmic mandibular movements while standing or lying; (c) *Walking*: slow locomotion related to exploration or patch selection; (d) *Social interaction*: affiliative behaviors such as allogrooming or gentle approaches; (e) *Resting*: recumbency in relaxed postures without signs of tension.2) Negative / Discomfort-Related Behaviors. (f) Attempts to remove or displace the harness; (g) Agitation, avoidance, excessive locomotion; (h) Muscular stiffness or guarded posture; (i) Reductions in grazing or rumination; (j) Social withdrawal. The complete absence of these behaviors across sessions was interpreted as evidence of behavioral neutrality of the harness.

### Behavioral recording and data management

For every 2-minute scan, the observer recorded: The number of animals expressing each behavior in the ethogram (*Count_total*). The number of those animals that were wearing the harness (*Count_vest*). And the remaining animals performing the behavior (*Count_noVest*).

### Statistical analysis

Statistical analyses were performed to determine whether the use of a harness to measure heart rate variability (HRV) changed the number of cows exhibiting specific behavior in the ethogram. Since the behavioral outcomes represented time-series count data, a generalized linear mixed model (GLMM) with a Poisson distribution was applied.

For each behavior, the response variable was the number of cows observed performing specific behavior during each scan. The fixed effect of interest was the harness condition (with or without a vest). A hierarchical structure was included in the model due to repeated measures, with sampling session (10 samplings) and scans (20 scans per session) included as intercepts. This structure controlled for temporal autocorrelation and daily variability in group activity. The model assumptions were verified by evaluating the dispersion parameters and residual patterns. The estimated coefficient for harness condition was interpreted as the multiplicative change in the rate of behavior associated with vest use. Descriptive statistics (means, medians, and proportions) and time-series visualizations were used to contextualize the model results.

## Results

**Fig. 1:**
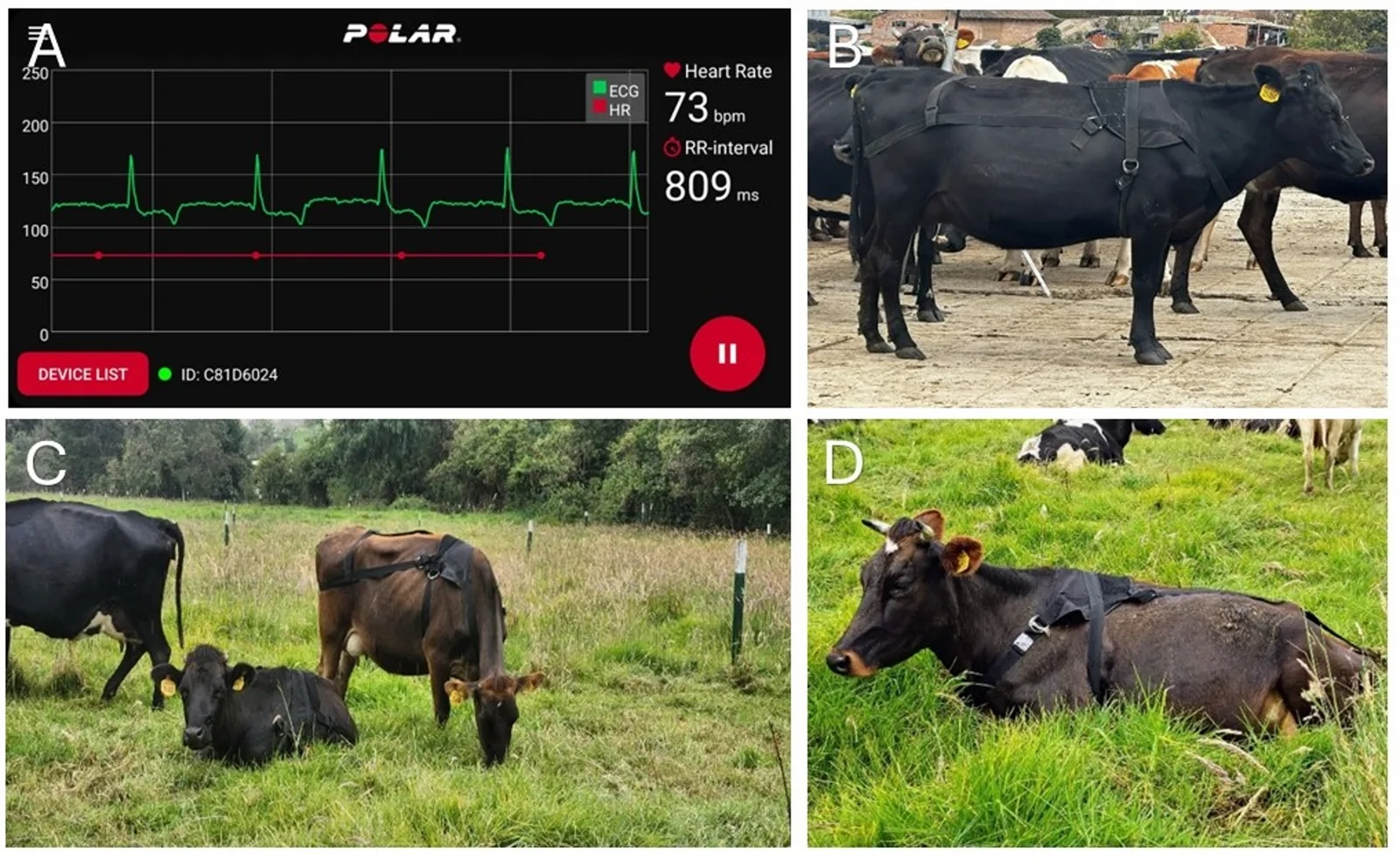
Vest-type textile design for monitoring remote heart rate variability (HRV) in grazing dairy cows under high-tropics conditions. **A**) Real-time heart rate monitoring interface obtained through the Polar Flow^®^ App connected to the Polar H10^®^ Holter-type sensor, illustrating on-screen ECG and interbeat interval (IBI) visualization during field use. **B**) Adult lactating dairy cow fitted with the vest-type harness showing correct dorsal stabilization, lateral strap adjustment, and secure placement of the thoracic sensor under grazing-farm conditions. **C–D**) Normal grazing and rumination behaviors observed in dairy cows wearing the vest in open pasture. The vest remained firmly positioned without restricting locomotion, postural changes, lying, or rumination cycles, demonstrating mechanical stability and behavioral compatibility during free movement

A total of 74 lactating dairy cows were evaluated during the study, organized into 10 observation sessions corresponding to each deployment of the HRV harness. The vest-type textile piece designed for this purpose remained stable throughout its use, as illustrated in the images showing the ECG interface with RR interval detection (Fig. 1A), the proper dorsal and thoracic placement of the harness on standing cows (Fig. 1B), and its compatibility with grazing, lying, and rumination postures in the field (Fig. 1C–D). The ethogram-based behavioral assessment demonstrated that cows adapted rapidly to wearing the harness and showed no negative behaviors across any of the 200 scan observations. No cow attempted to remove the vest, and no agitation, avoidance, stiffness, or disruptions in grazing or rumination were detected. During the first 10 min of each session, cows not wearing the vest displayed brief exploratory behaviors toward the vested individuals, including sniffing and licking the harness; after this, the entire herd resumed uninterrupted grazing. These exploratory interactions were transient and did not result in displacement, aggression, or changes in group cohesion.

**Fig. 2:**
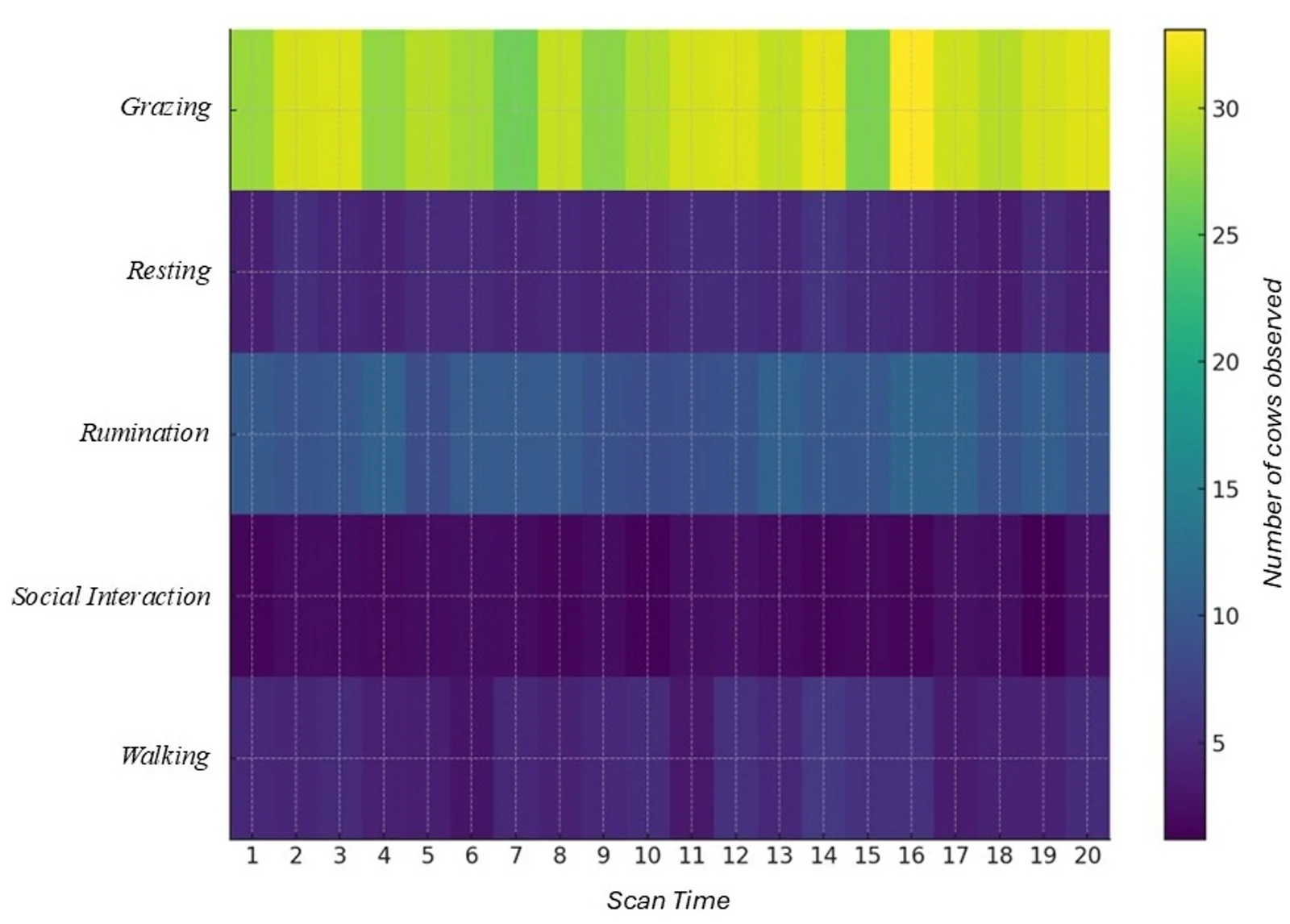
Kubios software heart rate variability analysis for a continuous 5-hour HRV recording in an adult lactating dairy cow under high-altitude tropical grazing conditions. **A**) Time-series plot of heart rate (HR) variations after automated correction of abnormal interbeat intervals (> 30%), following the correction guidelines reported by Kovács et al. (2015a, b). Abnormal or ectopic intervals commonly caused by sudden movements or motion artifacts—were automatically detected and removed to ensure accurate HRV parameter extraction. **B**) HRV dynamics over the same recording period, illustrating temporal fluctuations in autonomic nervous system activity. Parasympathetic modulation (blue trace) and sympathetic modulation (orange trace) are shown across the recording, highlighting typical autonomic patterns in grazing dairy cows under non-restrictive field conditions

**Fig. 3:**
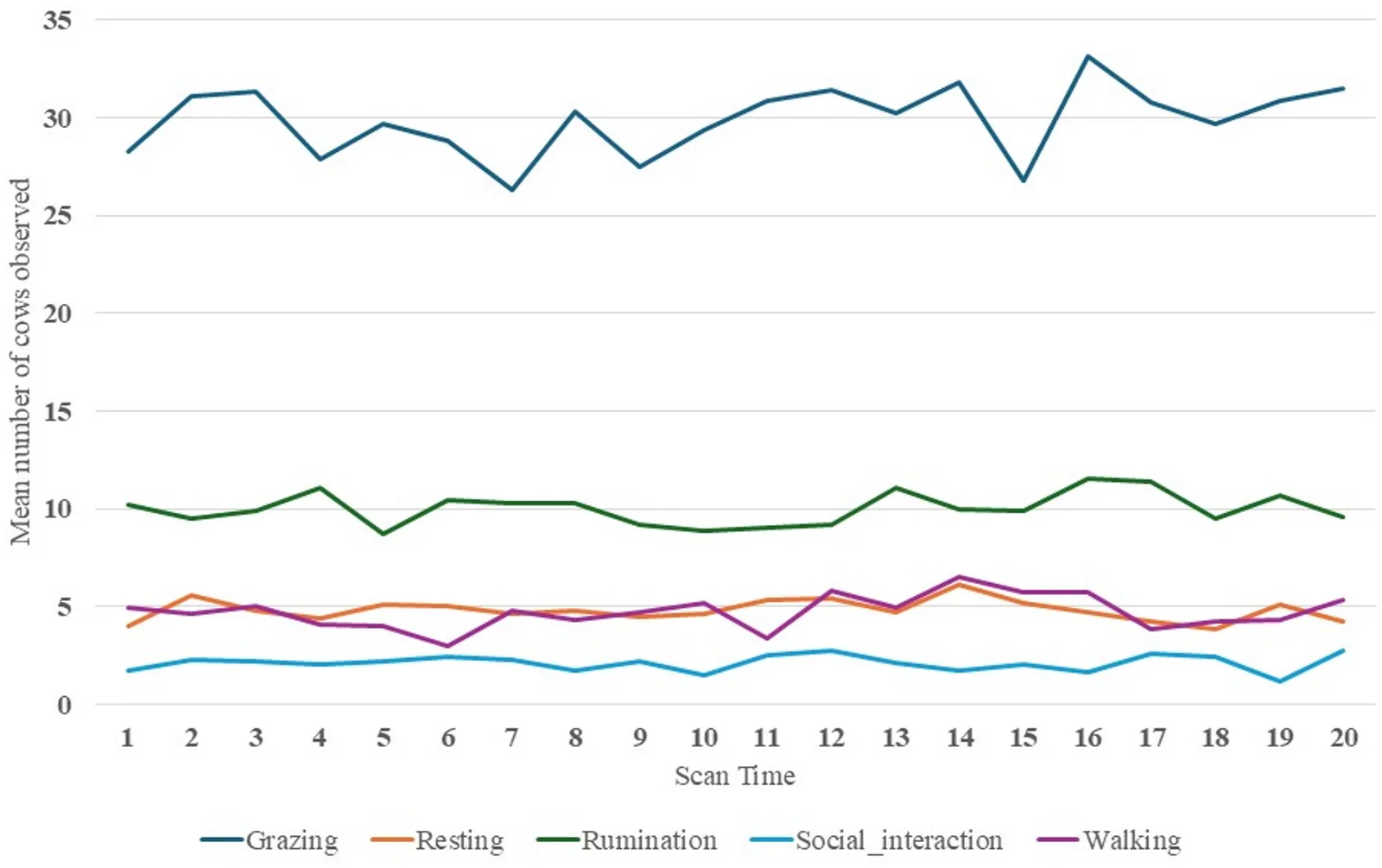
Heatmap of mean behavioral occurrence across observation sessions in an adult lactating dairy cow (*n* = 74) under high-altitude tropical grazing conditions. Heatmap illustrating the mean number of cows expressing each behavior (grazing, rumination, walking, resting, and social interaction) across all 20 live-scan observations per session. Warmer colors indicate higher behavioral occurrence, whereas cooler colors represent low-frequency expressions. Grazing exhibited the greatest and most consistent intensity throughout all scans, followed by rumination, with the remaining behaviors occurring at lower levels. No abrupt fluctuations or anomalies were observed, indicating stable behavioral expression across sessions and confirming that the presence of the HRV vest did not modify the normal behavioral repertoire of cows under rotational grazing conditions

**Fig. 4:**
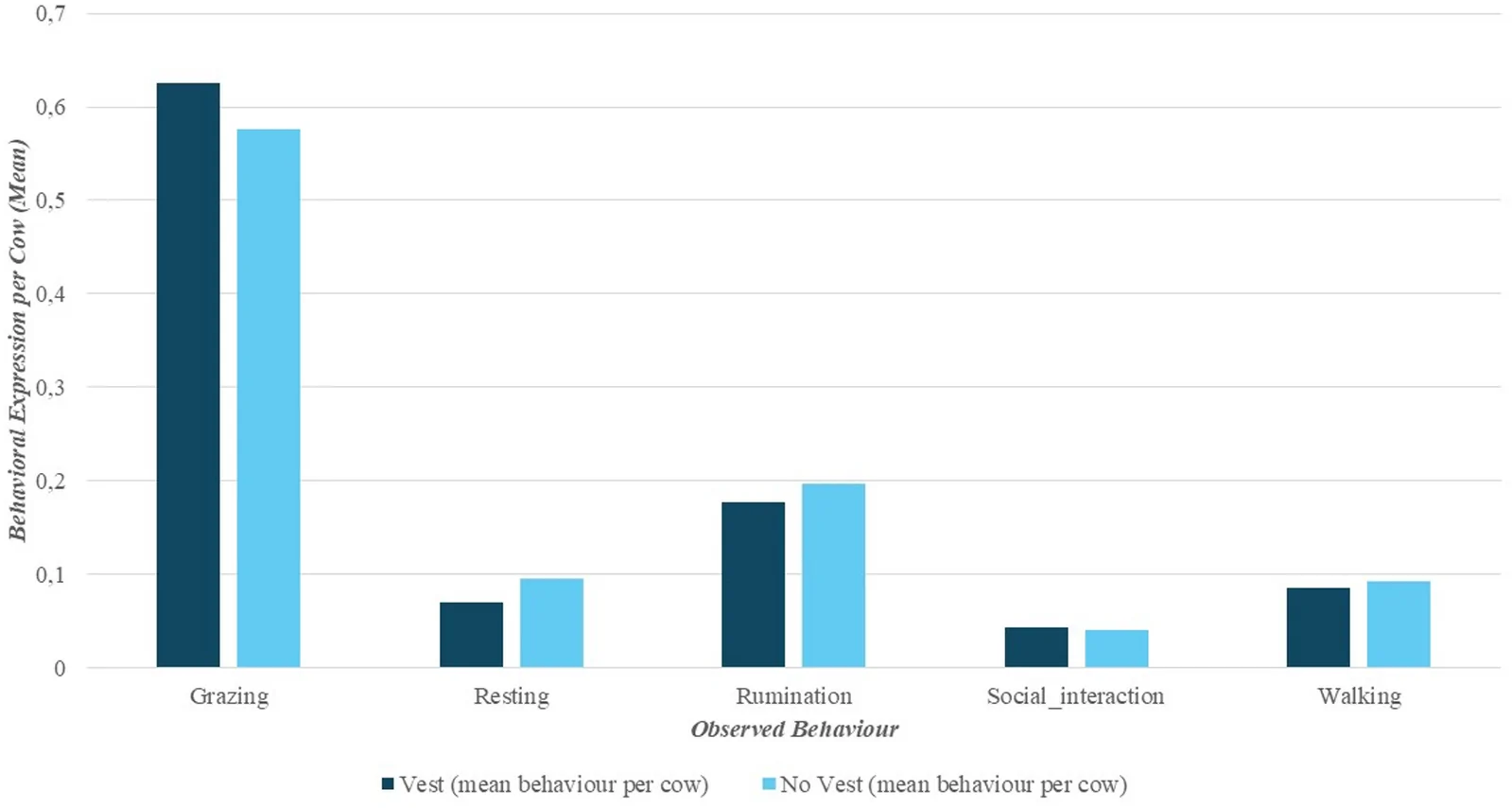
Multicomponent time-series of mean behavioral expression across live-scan observations in lactating dairy cows (*n* = 74) under grazing conditions. Time-series visualization showing the temporal progression of the mean number of cows engaged in the five ethogram behaviors across the 20 scans averaged over all sessions. Grazing consistently remained the dominant behavior throughout the observation period, while rumination exhibited moderate fluctuations typical of mid-day grazing patterns. Walking, resting, and social interaction occurred at low but stable frequencies, without abrupt peaks or declines. The relative separation of curves for each behavior reflects a well-defined and stable behavioral hierarchy, with no evidence of behavioral disruption associated with the use of the HRV vest

Across all sessions, the most frequently expressed behavior was grazing, followed by rumination, with walking, resting, and social interaction appearing less frequently. The heatmap of mean behavioral expression (Fig. 2) showed a stable pattern across scans, with grazing consistently ranging between approximately 27 and 33 cows per scan, rumination between 8 and 12, and lower-frequency behaviors (walking, resting, social interaction) remaining within narrow, low-intensity ranges. These trends were confirmed by the time-series analysis (Fig. 3), where grazing and rumination maintained dominant and consistent expression over time, without abrupt peaks or declines. Behavioral mean counts for vest vs. non-vest cows were proportional to their representation in the herd (Fig. 4) and did not show differences in behavioral mean comparison. Counts of grazing and rumination were highest for both groups, while resting, walking, and social interaction remained less frequent, as expected under mid-day grazing conditions. These results confirm that the presence of the harness does not interfere with locomotion, feeding, rumination, social behavior, or resting.

Table 1 shows the results of the Poisson generalized linear model evaluating the effects of behavior type, use of vest (vest vs. no-vest), and observation time on the number of behavioral events recorded across behavior observation scans. A significant effect of group was detected, with cows wearing the vest showing lower behavioral event counts compared with control cows (β=–2.41, *p* < 0.001). However, neither the effect of time nor the group × time interaction reached statistical significance (*p* = 0.075 and *p* = 0.883, respectively), indicating that behavioral expression remained stable across observation scans and that the pattern over time did not differ between Vest and No Vest animals.

**Table 1:** Poisson generalized linear model evaluating the association of behavior type, vest utilization, and behavior scan time on behavioral event counts in lactating dairy cows (*n* = 74) grazing in highland tropics

Variable	Coefficient	Std. Error	z-value	*p*-value	95% Confidence Interval	
					Lower	Upper
Grazing	Ref.	-	-	-	-	-
Resting	-1.8277	0.035	-52.59	< 0.001	-1.896	-1.760
Rumination	-1.0928	0.026	-42.33	< 0.001	-1.143	-1.042
Social interaction	-2.6554	0.050	-52.60	< 0.001	-2.754	-2.556
Walking	-1.8477	0.035	-52.70	< 0.001	-1.916	-1.779
Vest	-2.4121	0.075	-32.06	< 0.001	-2.560	-2.265
Time Scan	0.0032	0.002	1.78	0.075	-0.000	0.007
Group × Time	-0.0009	0.006	-0.148	0.883	-0.013	0.011
Intercept	3.2788	0.023	141.96	< 0.001	3.234	3.324

**Fig. 5:**
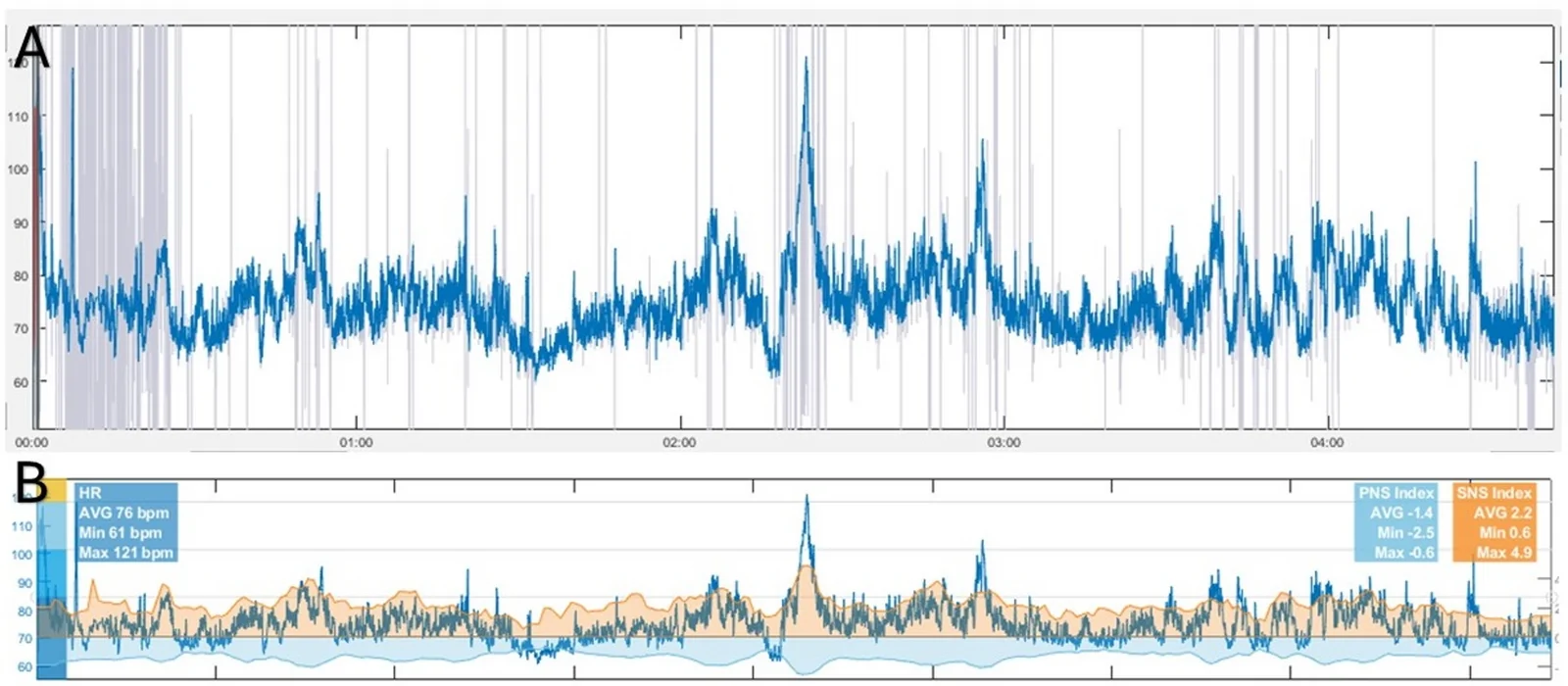
Bar graph of mean behavioral counts comparison in cows wearing the HRV vest versus cows without the vest across all observation sessions. Data represent the average proportion of cows exhibiting each behavior across all scan sessions, standardized by the number of cows observed in each group. Overall, grazing was the predominant behavior in both groups, with similar average expression between Vest (0.63) and No Vest (0.58) animals. Resting and rumination also showed comparable patterns between groups, with slightly higher values for No Vest cows (0.10 and 0.20, respectively) compared with Vest cows (0.07 and 0.18). Social interaction was low and nearly identical between groups, while walking exhibited minimal differences (Vest: 0.09; No Vest: 0.09). No differences were found between vest and non-vest cows

Regarding HRV collection, the harness allowed continuous remote ECG and RR interval acquisition under free-grazing conditions. Recording durations varied across individuals, with 18.9% of cows providing 1–2 h of usable recording, 28.4% providing 3–4 h, and 52.7% providing 5–7 h of data. Representative ECG and time-series RR interval outputs are shown in Fig. 5(A-B). For HRV analysis, the first five minutes following release into the pasture and the last five minutes before gathering the animals were removed to eliminate handling-related artifacts. All recordings were processed using Kubios Scientific software with standard threshold-based artifact correction, ensuring the extraction of clean NN intervals. HRV evaluation included linear, frequency-domain, and nonlinear parameters, with summary values for grazing and rumination shown in Table 2. In the animals, RR intervals increased during rumination relative to grazing, and RMSSD and SDNN values fell within ranges characteristic of relaxed cattle. LF/HF ratios remained low, and nonlinear indices (SD2/SD1) exhibited stable fractal dynamics, consistent with physiological regulation under low-arousal grazing conditions.

**Table 2:** Reference values for several HRV parameters related to frequency, linear, and nonlinear domains for lactating dairy cows (*n* = 74) grazing in Highland tropics

HRV parameters	Mean ± SD	HRV Range	90% CI
MRR*	766.6 ± 112.5	545.9–987.2	533.2–997.8
SDNN**	24.0 ± 14.1	7.5–45.8	7.3–45.8
RMSSD**	12.0 ± 8.9	3.5–35.4	3.3–38.1
LF/HF**	11.3 ± 8.9	1.3–34.4	1.2–36.0
SD2/SD1*	3.6 ± 1.1	1.4–5.8	1.3–5.9

HRV = Heart rate variability; MRR = Mean RR interval (ms); SDNN = Standard deviation (SD) of the NN interval (m/sec); RMSSD = Root mean square of the successive differences (m/sec); LF/HF = ratio of low frequency (LF) to high frequency (HF); SD2/SD1 = Relation between the SD of the Poincare plot along the line of identity (SD2) and the SD of the Poincare plot perpendicular to the line of identity (SD1). * Normal distribution. ** Box‒Cox transformed data

## Discussion

Sensors can capture accurate physiological data, reducing human intervention, which is an advantage from an ethological point of view since an animal can express its natural behavior (Stewart et al. 2005). Currently, Holter-type sensors are among the most practical methods for HRV measurement (Chalmeh and Karamifar 2021); however, in environments such as high-tropic grazing, the use of Holter sensors is complex since the sensors can easily lose contact with the animal and lose the information provided by the records (Grigg et al. 2021). Monitoring the HRV in cattle through sensors is especially beneficial if the operator ensures that the sensor has direct contact with the animal’s skin. This is difficult in bovines because the skin is thick and can make contact difficult (Pérez-Hernández et al. 2024). This study presents an innovative option for the use of Holter-type sensors for the constant and reliable measurement of HRV via a vest that guarantees HRV recording without changes in grazing behavior or the daily routines of the animal. Noninvasive alternatives to measure physiological parameters in cows grazing in the high tropics have significant advantages, especially for evaluating animal welfare, where stress responses that occur with invasive methods are minimized (Schaefer et al. 2012; Scoley et al. 2019).

The HRV allows the evaluation of the balance between the sympathetic and parasympathetic systems, providing an indirect measure of stress and the animal’s general well-being (Konold et al. 2011). In this context, sensors that record HRVs without interfering with cows’ activities under high tropical grazing conditions allow continuous, real-time monitoring of their physiological status (Wierig et al. 2018). This long-term monitoring capacity is essential for identifying long-term patterns that could go unnoticed in short-term studies or those that rely on single measurements. Therefore, ensuring the conduction of information is vital to determine the veracity of possible changes that reflect positive or negative animal emotional states (Kahankova et al. 2022; Mohr et al. 2002). The vest-type textile piece guarantees that the sensors and the watch are covered and allows the proper conduction of cardiac information. The design of the vest did not cause physical discomfort or friction that could lead to animal skin injuries, and its fit did not restrict natural movements. In the animals, RR intervals increased during rumination relative to grazing, and RMSSD and SDNN values fell within ranges characteristic of relaxed cattle. LF/HF ratios remained low, and nonlinear indices (SD2/SD1) exhibited stable fractal dynamics, consistent with physiological regulation under low-arousal grazing conditions (Fig. 5).

Compared with previous studies evaluating Holter-based cardiac monitoring in domestic animals, the present work expands the applicability of HRV measurement into conditions of far greater environmental and behavioral complexity. Chalmeh and Karamifar (2021) demonstrated that both short-term electrocardiography and 24-hour Holter monitoring can reliably detect cardiac electrical activity and arrhythmias in housed Holstein cows, but their recordings were obtained under controlled, low-movement environments that inherently favor electrode stability. Similarly, Ogawa et al. (2022) validated patch Holter devices in healthy indoor cats, where movement restrictions and environmental uniformity minimized artifact generation and ensured stable signal conduction. Likewise, Huangsaksri et al. (2024) successfully recorded HR and HRV in horses undergoing hot and cold shoeing procedures performed under supervised conditions with animals restrained or minimally mobile. In contrast, the current study demonstrates that sustained, high-quality HRV recordings are feasible in dairy cows under unrestricted, high-tropic grazing conditions, where posture changes, locomotion, vegetation contact, and weather fluctuations present substantial challenges to electrode stability.

These results demonstrate that the vest-type harness: (1) remains mechanically stable under pasture conditions, (2) does not induce behavioral alterations or discomfort detectable through ethogram-based assessment, (3) permits animals to perform all natural behaviors including lying and rumination, (4) supports multi-hour continuous HRV recording in more than half of deployed cases, and (5) produces analyzable RR interval data adequate for time-, frequency-, and nonlinear-domain HRV metrics. The vest-type textile system used here enabled reliable HRV capture despite these field constraints, highlighting a methodological advancement that bridges the gap between controlled-environment Holter research and real-world physiological monitoring in free-ranging cattle. Within the limitations of the vest, there were variations in the recording times, and we hypothesize that unusual movements of the cows or the weather may be related to changes in the recording times. Although uncommon, highly abrupt movements of the animal can affect sensor conduction and recording times. Unlike dry periods, rainy periods favor the sensor belt to remain wet, improving conduction efficiency. In all cases, especially when HRV recording is associated with animal comfort and welfare measurements, HRV measurement must be accompanied by a previous clinical examination, echocardiogram, and BGME analysis. Future research related to elements for vest use improvement, methodologies for longer recording times, and the detection of factors affecting the quality of HRV measurement is necessary. This tool provides the opportunity to explore different research topics associated with HRVs, such as dairy cattle animal welfare and comfort, and bovine internal medicine.

## Limitations

This study presents several limitations that should be considered when interpreting the findings. Environmental variables such as temperature, humidity, rainfall, and solar exposure were not recorded, even though they likely influence electrode skin conduction and sensor stability under grazing conditions. Individual cow-level characteristics (age, body size, production level, rumen fill, or skin thickness) were not included in the analyses, and these factors may affect autonomic responses and recording continuity. Although no dermatological alterations were observed, a formal skin integrity scoring protocol was not implemented, limiting the capacity to objectively evaluate dermal tolerance to prolonged vest use. Additionally, while observations suggested that abrupt movements and certain weather conditions coincided with signal interruptions, these associations were descriptive rather than quantitatively tested. The absence of a direct comparison with a validated medical-grade Holter device also restricts conclusions about measurement accuracy across devices. Future research should explore cross-validation studies, controlled environmental manipulations, standardized dermatological assessments, and larger sample sizes to refine the vest’s performance and better characterize the determinants of HRV recording quality in free-grazing cattle.

## Conclusions

The vest-type textile system developed in this study demonstrated strong potential as a field-adapted, behaviorally neutral, and reliable tool for HRV monitoring in dairy cows under high-tropic grazing conditions. By stabilizing the sensor, preserving electrode contact, and maintaining compatibility with natural grazing, social, and resting behaviors, the vest enabled extended HRV recordings that reflect true autonomic activity rather than handling or equipment artifacts. Although environmental variables and cow-specific characteristics were not assessed, and no gold-standard comparison was performed, the vest consistently supported multi-hour HRV data acquisition under conditions where traditional belts or adhesives often fail. These findings highlight the vest’s value for advancing welfare monitoring, behavioral ecology research, and clinical assessments in extensive livestock systems. Future studies should expand on these results by integrating environmental measures, validating the system against medical-grade Holter devices, and evaluating performance across different management systems to fully establish the vest as a robust platform for precision physiological monitoring in cattle.

## Supplementary Information

Below is the link to the electronic supplementary material.


Supplementary Material 1


## Data Availability

The data that support the findings of this study are available from the corresponding author upon reasonable request.
